# Three new species of *Levinsenia* Mesnil, 1897 (Annelida: Paraonidae) from shallow and deep waters of Southwestern Atlantic Ocean

**DOI:** 10.1371/journal.pone.0244741

**Published:** 2020-12-31

**Authors:** Natália Ranauro, Rômulo Barroso, João Miguel de Matos Nogueira

**Affiliations:** 1 Laboratório de Poliquetologia, Departamento de Zoologia, Instituto de Biociências, Universidade de São Paulo, São Paulo, SP, Brazil; 2 Laboratório de Annelida, Instituto de Biologia, Universidade Federal da Bahia, Salvador, BA, Brazil; CIIMAR Interdisciplinary Centre of Marine and Environmental Research of the University of Porto, PORTUGAL

## Abstract

Three new species of *Levinsenia* were collected during a benthic survey, from 10–3,000 m deep, in Espírito Santo Basin, off the southeastern Brazilian coast. These species are *L*. *paivai* **sp. nov.**, *L*. *blakei* **sp. nov.** and *L*. *lesliae* **sp. nov.** Members of *L*. *paivai* **sp. nov.** are recognized by the presence of nine pairs of well-developed and heavily ciliated branchiae, those of *L*. *blakei* **sp. nov.** are characterized by the presence of three pairs of small branchiae, and those of *L*. *lesliae* **sp. nov.**, by the absence of branchiae and presence of notopodial transitional chaetae. These new species are described herein and compared to the most similar congeners. These are the first new species of *Levinsenia* described from off the Brazilian coast.

## Introduction

Among the annelids inhabiting soft substrates, individuals of the family Paraonidae Cerruti, 1909 [1] are among the more abundant in shelf and slope depths [2–4]. This family has extraordinarily high species richness in deep-water habitats [5, 6] although a few species are also found in intertidal environments [7]. Paraonids are small burrowing worms, mostly between 2–4 cm long and 0.1–0.2 mm wide [2].

The family Paraonidae currently includes about 155 described species, with a worldwide distribution [8, 9]. Due to the utilization of finer (0.5 mm mesh) sieves and the numerous projects focused on diversity in deep-sea environments [10], several new species have been described recently [11–13].

The genus *Levinsenia* Mesnil, 1897 [14] (25 sp.) is the third most diverse genus of the family, with several species described in the last two decades [5,15–18]. The first described species of *Levinsenia* was *Aonides gracilis* Tauber, 1879 [19], which was originally placed in the family Spionidae Grube, 1850 [20]. The genus *Levinsenia* was erected by Mesnil [14], into which the author allocated *A*. *gracilis* and *A*. *fulgens* Levinsen, 1884 [21]. The family Levinseniidae was erected by Mesnil & Caullery [22], to accommodate the genera *Levinsenia* and *Aricidea* Webster, 1879 [23]. However, these authors did not include *Paraonis tenera* Grube, 1873 [24], which was the first Paraonidae described and was placed by the time in the family Spionidae. Later, Cerruti [1] erroneously synonymized *Levinsenia* with *Paraonis* and consequently renamed the family Levinseniidae to Paraonidae [2].

Strelzov [2], in a major review of the Paraonidae, noticed remarkable differences among specimens of the species assigned to *Paraonis*. The author kept *P*. *fulgens* (Levinsen, 1884) [21] and *P*. *pygoenigmatica* Jones, 1968 [25] in *Paraonis*, and split the remaining species among *Sabidius* Strelzov, 1973 [26] and *Tauberia* Strelzov, 1973 [26], members of *Sabidius* having a prostomium with a trilobate anterior margin, while the animals belonging to *Tauberia* have cirriform notopodial postchaetal lobes. *Tauberia* was considered as a synonym of *Levinsenia*, according to the rules of the ICZN [5].

Members of *Levinsenia* are abundant in shelf and slope depths, although some species can also be found in shallow waters [27]. The external morphology of these animals is remarkably simple, in comparison to those belonging to the other genera in this family, and therefore there is a limited number of diagnostic characters [2], but all *Levinsenia* have: (1) prostomium lacking antenna; (2) presence of terminal sensory organ (palpode) on prostomium; (3) when branchiae are present, there are always more than 4 prebranchial segments; (4) pygidium tapering, with two anal cirri. Also, the use of modern equipment, such as the Scanning Electron Microscope (SEM), has been very useful in the search for new morphological characters, as well as the methyl green staining, which provided useful species specific staining patterns [16, 18].

Specimens of *Levinsenia* have been found in all oceans. At least some species of the genus have been reported for polar regions, one for the Arctic Ocean and two for the Southern Ocean. In the Pacific, 12 species have been reported, seven for the northern Pacific and five for the southern. The Atlantic Ocean has the highest number of species of for the genus, with 10 species reported from the northern Atlantic and five from the southern [5]. Regarding the Brazilian coast, previous studies on paraonids registered the presence of six genera and 38 species, most of them from shallow waters [28]. Few systematic studies have been conducted on Brazilian paraonids [11, 29–31], so most (62%) of the previous records for the family in Brazil come from gray literature and papers with an ecological approach, in which specimens descriptions and illustrations are not given [28]. For this reason, most of these records are questionable.

The aim of this study is to describe three new species of *Levinsenia* based on specimens collected from off southeastern Brazil, state of Espírito Santo, between 19°3'S 37°44'W and 21°10'S 38°28'W. Out of the 38 species of paraonids previously reported for the Brazilian coast, three belong to *Levinsenia* [28], although the reliability of this records is questionable, as discussed above. So the present paper raises to six the number of species of this genus occurring off Brazil.

## Material and methods

The specimens were collected during a project (AMBES—Environmental Characterization of the Espírito Santo Basin and North of the Campos Basin) conducted by CENPES/PETROBRAS (Brazilian Energy Company). Soft bottoms samplings were collected in the Southwestern Atlantic, off the Espírito Santo State, from December 2010 to July 2013, between 19°3'S 37°44'W and 21°10'S 38°28'W, Southeastern Brazil. As CENPES/PETROBRAS are a Brazilian government company no specific permissions were required to do the sampling. Field studies did not involve endangered or protected species.

**Fig 1:**
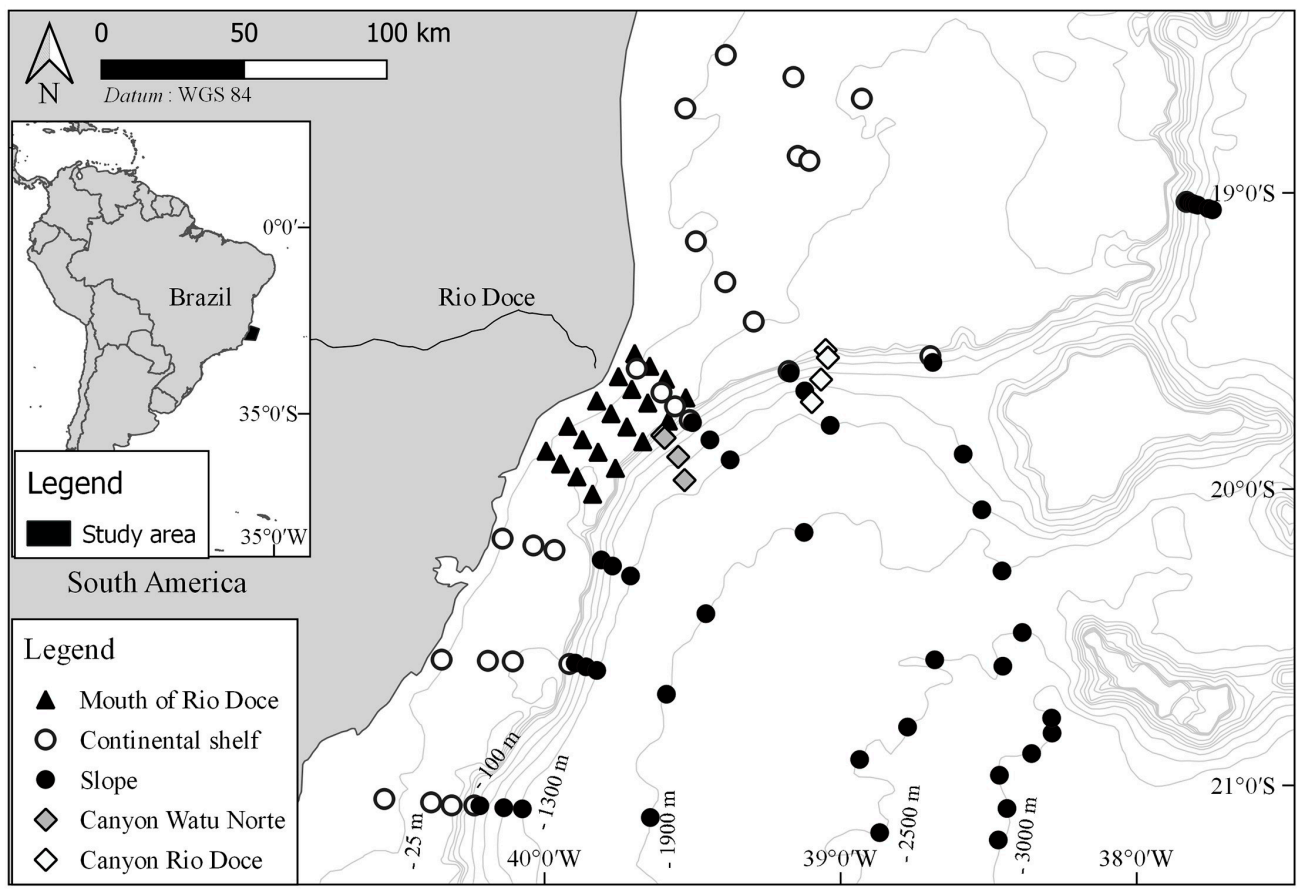
Study area. Sampling stations at four different areas: Mouth of Rio Doce, continental shelf, slope and canyons.

Field sampling was performed on board of the oceanographic vessels Seward Johnson and Luke Thomas. Sampling was focused on four soft bottoms environments: mouth of Rio Doce (20 stations, 10–51 m deep), continental shelf (28 stations, 25–150 m), slope (42 stations, 400–3,000 m) and two different canyons (canyon Rio Doce and canyon Watu Norte) (4 stations each, both 150–1,300 m) (Fig 1). Each of these four environments was sampled twice, in summer and again in winter. Samples were collected in three replicates at each station, using either a 294 L van Veen grab or a 125 L box-corer. The sediment was then sieved in a 0.5 mm mesh, fixed in 10% sea-water formalin, and preserved in 70% ethanol.

Identifications were based on specific morphological characters. Specimens were examined using a stereomicroscope, compound light microscope and scanning electron microscope (SEM). For the SEM, specimens were dehydrated in a series of progressively stronger concentrations of ethanol (70–100%), then critical point dried (LEICA EM CPD300), coated with ~35 nm of gold and examined and photographed at the Laboratório de Microscopia Eletrônica, Instituto de Biociências, Universidade de São Paulo (IB–USP). Also, some specimens were stained with methyl green, to investigate for species specific staining patterns.

Descriptions were made based on the holotypes and variation observed among paratypes is provided inside parentheses, after each corresponding measurement of the holotype. Complete specimens were measured; width was always taken at chaetiger 4. Maps were created using the Free and Open Source QGIS [32].

The following institutional abbreviations are used: LACM–AHF, Allan Hancock Foundation Polychaete Collection, Natural History Museum of Los Angeles County, Los Angeles, USA; MZUSP, Museu de Zoologia da Universidade de São Paulo, São Paulo, Brazil; ZUEC, Museu de Zoologia, Universidade Estadual de Campinas, Campinas, Brazil; UFBA, Museu de História Natural da Bahia, Salvador, Brazil. Specimens are deposited in MZUSP; ZUEC; UFBA.

### Nomenclatural acts

The electronic edition of this article conforms to the requirements of the amended International Code of Zoological Nomenclature, and hence the new names contained herein are available under that Code, from the electronic edition of this article. This published work and the nomenclatural acts it contains have been registered in ZooBank, the online registration system for the ICZN. The ZooBank LSIDs (Life Science Identifiers) can be resolved and the associated information viewed through any standard web browser by appending the LSID to the prefix “http://zoobank.org/”. The LSID for this publication is: urn:lsid:zoobank.org:pub:2BBA7519-EFED-4B39-A67A-AE3CF11765FA. The electronic edition of this work was published in a journal with an ISSN and has been archived and is available from the following digital repositories: PubMed Central, LOCKSS.

## Results

### Systematics

#### Family Paraonidae Cerruti, 1909

*Genus Levinsenia Mesnil*, *1897*. Type species. *Aonides gracilis* Tauber, 1879 [19], designated by ICZN [33].

*Description*. Body long and thin, threadlike. Prostomium lacking antenna; with terminal sensory organ (palpode); prostomial ciliary bands absent; nuchal organs along posterior prostomial margin; lateral sensory organs (cheek organs) present or absent, on sides of prostomium; prostomial ciliary patches present or absent. Prebranchial segments numbering 4–8; number of branchial segments variable (3–36 pairs), branchiae absent in some species. All notopodia with postchaetal lobes; neuropodial postchaetal lobes absent. Notochaetae all capillaries; neurochaetae include capillaries and thick modified spines; spines often curved, with distinct fringe on convex side. Pygidium tapering, with two anal cirri [5, 18].

**Fig 2:**
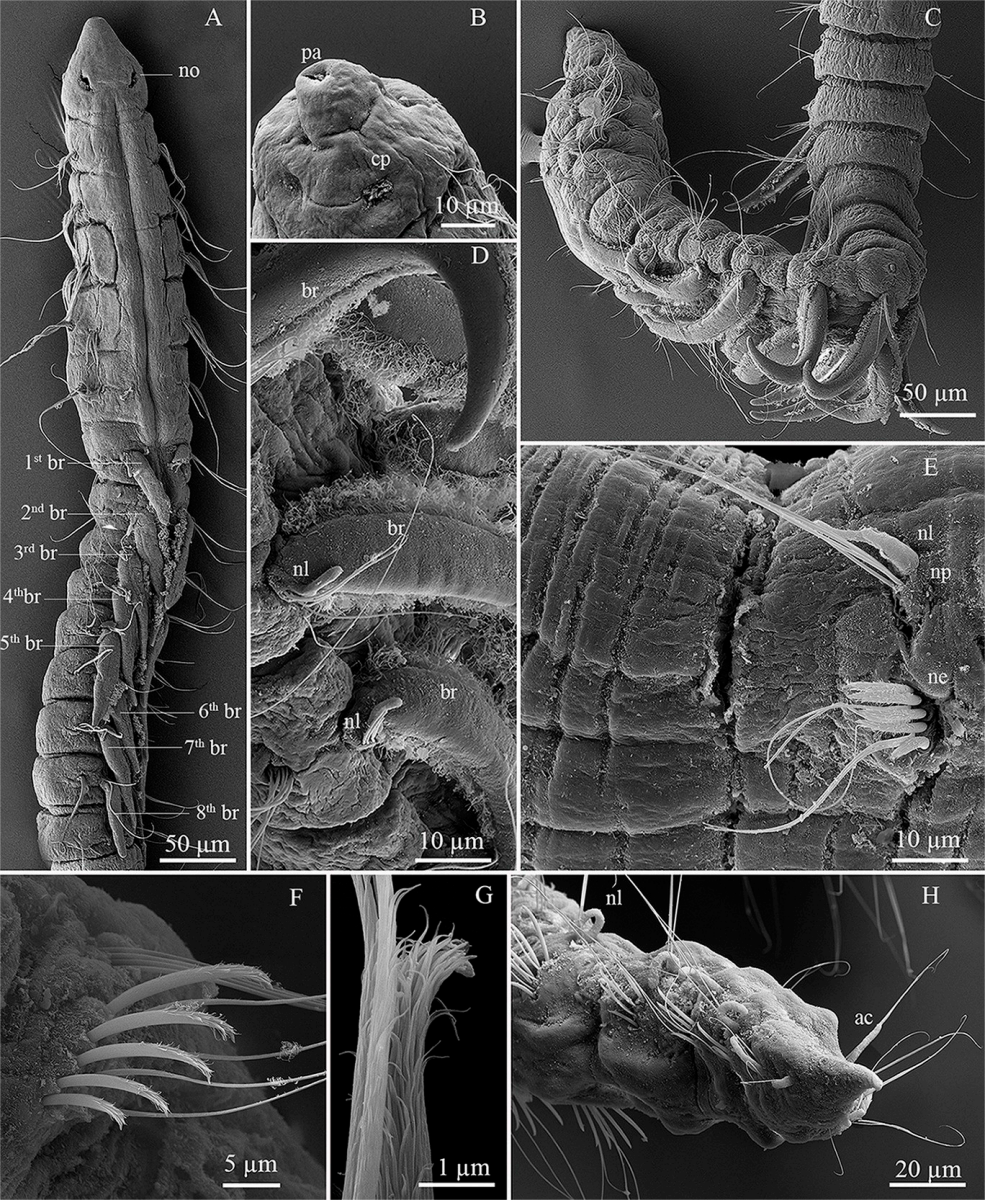
*Levinsenia paivai* sp. nov. (A). Anterior end, dorsal view; (B). Prostomium, frontal-ventral view; (C). Branchial chaetigers, dorsal–lateral view; (D). detail of branchiae, lateral view; (E). Parapodium, chaetiger 22, lateral view; (F). Parapodium, chaetiger 44, lateral view; (G). Curved modified spine, chaetiger 47; (H). Posterior end with pygidium, lateral view. ac = anal cirrus; br = branchia; cp = ciliary patches; ne = neuropodium; nl = notopodial postchaetal lobe; no = nuchal organ; np = notopodium; pa = palpode.

**Fig 3:**
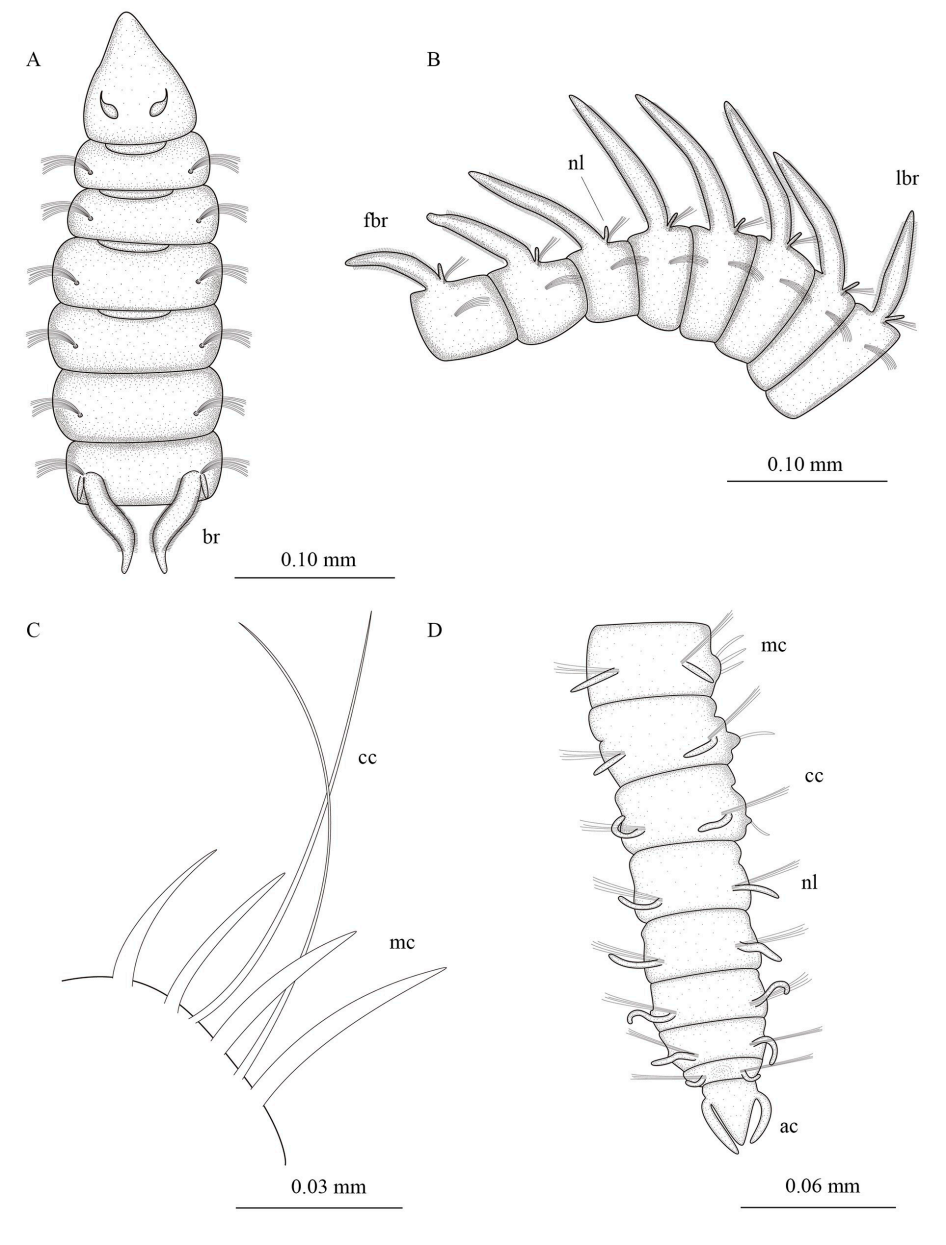
*Levinsenia paivai* sp. nov. (A). Anterior end, dorsal view; (B). Branchial segments, lateral view; (C). Neuropodium, chaetiger 68; (D). Posterior end, dorsal view. ac = anal cirrus; br = branchia; cc = capillary chaeta; fbr = first branchia; lbr = last branchia; mc = modified chaeta; nl = notopodial postchaetal lobe.

**Fig 4:**
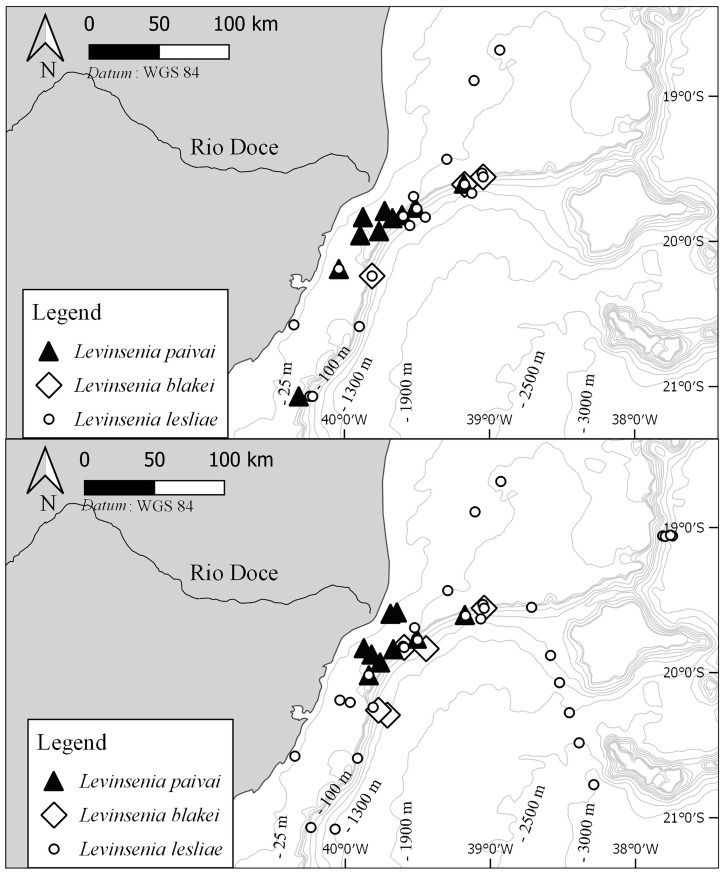
Sampling sites from which members of species of *Levinsenia* were obtained, in summer (above) and winter (below).

*Levinsenia paivai* sp. nov. (Figs 2–4)

urn:lsid:zoobank.org:act: 36E95A9C-41F6-4770-88A7-37FEFEE2D3F5

*Material examined*. *Type series*. Holotype (MZUSP–4131): 82 chaetigers, complete, 9.78 mm long, 0.14 mm wide, coll. 20 Jan 2012, 20°11'25.35"S 40°02'16.02" W, 35 m; Paratypes: 1 spec. (MZUSP–4132), coll. 16 Dec 2010, 19°55'44.66"S 39°45'38.7"W, 46 m; 3 specs. (MZUSP–4133), coll. 16 Dec 2010, 20°01'02.6"S 39°50'18.72"W, 49 m; 1 spec. (MZUSP–4134), coll. 16 Jul 2011, 19°55'45.59"S 39°45'41.35"W, 43 m; 3 specs. (MZUSP–4135), coll. 20 Jan 2012, 20°11'25.35"S 40°02'16.02"W, 35 m; 3 specs. (MZUSP–4136), coll. 15 Jan 2012, 19°45'54.56"S 39°30'25.23"W, 150 m; 2 specs. (MZUSP–4137), coll. 27 Jun 2013, 19°45'53.43"S 39°30'25.97"W, 138 m; 2 specs. (UFBA–1858), coll. 16 Dec 2010, 20°01'02.6"S 39°50'18.72"W, 49 m; 2 specs. (UFBA–1859), coll. 20 Jan 2012, 20°11'25.35"S 40°02'16.02"W, 35 m; 1 spec. (UFBA–1860), coll. 15 Jan 2012, 19°45'55.39"S 39°30'25.74"W, 121 m; 2 specs. (ZUEC–21455), coll. 16 Dec 2010, 20°01'02.6"S 39°50'18.72"W, 49 m; 2 specs. (ZUEC–21456), coll. 16 Jul 2011, 19°55'45.59"S 39°45'41.35"W, 43 m; 2 specs. (ZUEC–21457), coll. 20 Jan 2012, 20°11'25.35"S 40°02'16.02"W, 35 m; 1 spec. (ZUEC–21458), coll. 27 Jun 2013, 19°45'53.43"S 39°30'25.97"W, 138 m. Specimens mounted on SEM stubs: 3 specs. (ZUEC–21459), coll. 15 Dec 2010, 19°49'57.38"S 39°52'14.02"W, 29 m; 6 specs. (ZUEC–21460), coll. 27 Jun 2013, 19°45'53.43"S 39°30'25.97"W, 138 m.

*Additional material examined*. 2 specs. (MZUSP–4138), coll. 16 Dec 2010, 19°57'32.89"S 39°53'30.69"W, 43 m; 1 spec. (MZUSP–4139), coll. 14 Dec 2010, 19°50'22.01"S 39°40'06.1"W, 51 m; 1 spec. (MZUSP–4140), coll. 15 Dec 2010, 19°47'32.83"S 39°43'15.08"W, 37 m; 2 specs. (MZUSP–4141), coll. 15 Jul 2011, 19°50'16.39"S 39°40'11.23"W, 46 m; 1 spec. (MZUSP–4142), coll. 14 Jul 2011, 19°35'03.5"S 39°38'39.06"W, 30 m; 2 specs. (MZUSP–4143), coll. 16 Jul 2011, 19°52'29.66"S 39°49'08.1"W, 41 m; 1 spec. (MZUSP–4144), coll. 16 Jul 2011, 19°49'52.15"S 39°52'24.51"W, 28 m; 1 spec. (MZUSP–4145), coll. 14 Jan 2012, 19°49'07.27"S 39°36'08.52"W, 124 m; 2 specs. (MZUSP–4146), coll. 15 Jan 2012, 19°45'55.39"S 39°30'25.74"W, 121 m; 1 spec. (MZUSP–4147), coll. 15 Jan 2012, 19°36'04.32"S 39°10'34.07"W, 134 m; 1 spec. (MZUSP–4148), coll. 22 Jan 2012, 21°04'01.29"S 40°18'50.11"W, 46 m; 2 specs. (MZUSP–4149), coll. 29 Jun 2013, 19°36'03.57"S 39°10'33.64"W, 142 m; 1 spec. (MZUSP–4150), coll. 15 Dec 2010, 19°49'57.38"S 39°52'14.02"W, 29 m; 1 spec. (MZUSP–4151), coll. 16 Jul 2013, 19°35'35.96"S 39°41'20.61"W, 18 m.

*Comparative material examined*. *Levinsenia pycnobranchiata* (Fauchald, 1972) [34] Holotype LACM–AHF POLY 998, North Pacific, Mexico, Baja California, Gulf of California, 37 miles (bearing 060°T) from Punta Colorado, Isla San Jose, 25°22’00”N 109°59’00”W, 2,246 m, Campbell grab, R/V Velero, IV Sta. 11793–67, coll. Allan Hancock Foundation, 24 Nov 1967. *Levinsenia oculata* (Hartman, 1957) [35] Holotype LACM–AHF POLY 651, North Pacific, USA, California, Los Angeles County, outer Los Angeles harbor, 1 mile West (bearing 270°T) from Los Angeles Breackwater Lighthouse, 33°42’32”N 118°16’12”W, 7 Km, Rubbly clay, Hayward orange peel grab, R/V Velero IV, Sta. 2307–53, coll. Allan Hancock Foundation, 15 May 1953. *Levinsenia multibranchiata* (Hartman, 1957) [35] Holotype LACM–AHF POLY 647, North Pacific, USA, California, Santa Barbara County, Santa Barbara Channel Basin, 180 miles (bearing 135°T) from Point Conception Light, 34°14’10”N 120°12’45”W, 503 m, green mud, hayward orange peel grab, R/V Velero IV, Sta. 3731–55, coll. Allan Hancock Foundation, 12 Dec 1955.

*Description*. Holotype complete, with 82 chaetigers (54–78). Body 9.78 mm long (3.24–7.23 mm) and 0.14 mm wide (0.10–0.19 mm), fragile, easily broken. Anterior chaetigers dorso-ventrally flattened, twice wider than long (Figs 2A and 3A); from chaetiger 15 to posterior body, segments cylindrical, about as long as wide; last 8 chaetigers wider than long. Preserved specimens white–yellowish, without pigmentation patterns.

Prostomium conical, slightly longer [0.14 mm (0.08–0.14 mm)] than wide [0.10 mm (0.07–0.13 mm)] (Figs 2A and 3A); cylindrical terminal sensory organ (palpode) (Fig 2B), sometimes everted (0.04 mm long); cheek organs, eyes and median antenna all absent; patches of cilia present on ventral side (Fig 2B); pair of nuchal organs as upside down coma-shaped ciliated slits, on posterior margin of prostomium (Figs 2A and 3A). Posterior buccal lip with six longitudinal folds, extending to chaetiger 1. Ventral mouth, saclike pharynx everted in some specimens.

Prebranchial region with five chaetigers, each twice wider than long, chaetigers 4 and 5 widest; notopodial postchaetal lobes as short, rounded tubercles (Figs 2A and 3A). First pair of branchiae on chaetiger 6, nine (6–9) pairs of well-developed cirriform branchiae, each 0.04 mm wide at base, distally tapered, blunt tipped, ciliated laterally (Figs 2A, 2C and 2D). Pairs of branchiae progressively longer, except last two pairs, which are shorter (Figs 2C, 2D and 3B). Notopodial postchaetal lobes on branchial chaetigers cirriform, blunt tipped, 0.04 mm (0.03–0.05 mm) in length, 0.01 mm wide (Figs 2D and 3B). Neuropodial postchaetal lobes absent throughout. Tubercular notopodial postchaetal lobes on first two postbranchial segments; notopodial postchaetal lobes progressively longer and more cirriform on postbranchial chaetigers (Figs 2E and 3D) until end of body, 0.05 mm (0.04–0.06 mm) long, 0.01 wide, shorter on last chaetiger.

Parapodia biramous and poorly developed, notochaetae all capillaries emerging dorso-laterally. Neurochaetae located laterally. Prebranchial chaetigers with chaetae organized in two rows, each notopodium with nine chaetae, branchial segments with five notochaetae per fascicle, postbranchial chaetigers with four (Fig 2E).

Neurochaetae of prebranchial and branchial chaetigers all capillaries. Postbranchial region with two types of neurochaetae: capillary and modified curved spines, with distinct fringe on convex side, starting on chaetiger 19. Prebranchial chaetigers with 12 capillary neurochaetae, in two rows; branchial chaetigers with six capillary neurochaetae each; postbranchial chaetigers, until chaetiger 30, with up to four modified thick spines in posterior row, slightly curved, with distinct fringe on convex side, and four accompanying capillaries in anterior row (Fig 2E); from chaetiger 30 to posterior end, 4–7 modified spines and 2–4 accompanying capillaries (Figs 2F and 3C); accompanying capillary chaetae on postbranchial region slenderer than those of preceding regions. Posterior modified spines slightly longer and thinner than anterior ones (Fig 2G). Pygidium conical, slightly longer [0.03 mm (0.03–0.04 mm)] than wide [0.02 mm (0.02–0.03 mm)]; with one pair of anal cirri 0.04 mm long (0.02–0.04 mm) (Figs 2H and 3D).

*Methyl green staining pattern*. Prebranchial region staining limited to postchaetal areas; as solid band on branchial and 2–3 postbranchial chaetigers.

*Habitat*. Found in substrates with a high percentage of sand (63%), some amount of mud (29%) and a small percentage of pebbles (8%); water temperature 14–24°C; between 28–150 m deep.

*Distribution*. Southern Atlantic Ocean: Southeastern Brazil, off state of Espírito Santo, 28–150 m (Fig 4).

*Remarks*. *Levinsenia paivai* **sp. nov.** belongs to a group of species which members have branchiae starting on chaetiger 6. Also belonging to this group are *L*. *demiri* Çinar, Dagli & Acik, 2011 [16], *L*. *flava* (Strelzov, 1973) [26], *L*. *kantauriensis* Aguirrezabalaga & Gil, 2009 [10], *L*. *marmarensis* Çinar, Dagli & Acik, 2011 [16], *L*. *multibranchiata* (Hartman, 1957) [35], *L*. *oculata* (Hartman, 1957) [35], *L*. *oligobranchiata* (Strelzov, 1973) [26], *L*. *pycnobrachiata* (Fauchald, 1972) [34], and *L*. *tribranchiata* Çinar, Dagli & Acik, 2011 [20].

Individuals of *L*. *paivai* **sp. nov.** have branchiae much longer than those of members of *L*. *flava*, *L*. *kantauriensis* and *L*. *tribranchiata*, in which branchiae do not exceed 0.10 mm in length [2, 10, 16], while in members of *L*. *paivai* **sp. nov.** even the smallest pair of branchiae is longer than 0.10 mm (Table 1).

**Table 1:** Branchial length of type specimens of *Levinsenia paivai* sp. nov.

Branchiae	Holotype	Paratypes
1^st^ pair	0.14 mm	(0.13–0.27) mm
2^nd^ pair	0.18 mm	(0.14–0.31) mm
3^rd^ pair	0.22 mm	(0.15–0.34) mm
4^th^ pair	0.23 mm	(0.17–0.33) mm
5^th^ pair	0.21 mm	(0.17–0.32) mm
6^th^ pair	0.23 mm	(0.18–0.35) mm
7^th^ pair	0.25 mm	(0.18–0.36) mm
8^th^ pair	0.22 mm	(0.18–0.28) mm
9^th^ pair	0.19 mm	(0.16–0.19) mm

Individuals belonging to *L*. *multibranchiata* and *L*. *pycnobranchiata* have more than 20 pairs of branchiae [34, 35], while members of *L*. *paivai* **sp. nov.** have only 6–9 pairs.

Animals belonging to *L*. *paivai* **sp. nov.** also share similar morphology of branchiae with individuals of *L*. *demiri*. However, members of this latter species have strongly curved and slightly protruding modified spines [16], while among members of *L*. *paivai* **sp. nov.** such spines are slightly curved and strongly protruding.

Individuals of *L*. *marmarensis* also share with members of *L*. *paivai* **sp. nov.** similar number of pairs and morphology of branchiae, 7–8 pairs in members of *L*. *marmarensis*, 6–9 pairs in *L*. *paivai* **sp. nov.**, ciriform in both. However, branchiae are ciliated among members of *L*. *paivai* **sp. nov.** and smooth in members of *L*. *marmarensis*. In addition, members of *L*. *paivai* **sp. nov.** have branchiae 0.13–0.36 mm long, progressively longer to posterior pairs, except for last two pairs, while in individuals of *L*. *marmarensis* the branchiae have a similar length throughout, all pairs 0.24 mm long. Furthermore, members of both species also differ in regards to the modified spines, which are strongly curved in the latter species [16].

Members of *L*. *paivai* **sp. nov.** differ from those of *L*. *oculata* in regards to the morphology of notopodial postchaetal lobes on prebranchial chaetigers, which are cirriform and progressively elongate among specimens of *L*. *oculata*, while they are tubercular and poorly developed in specimens of *L*. *paivai* **sp. nov.** [35].

Finally, specimens of *L*. *oligobranchiata* share with members of *L*. *paivai* **sp. nov.** similar distribution (6–9 pairs, among members of both species) and morphology of branchiae. However, those animals differ in branchial size, as among members of the former species branchiae are short and of even length, 0.18 mm long throughout, while individuals belonging to *L*. *paivai* **sp. nov.** have branchiae progressively longer, except for the last two pairs. They also differ in regards to the shape of notopodial postchaetal lobes, which are distinctly short to inconspicuous on posterior segments, in individuals of *L*. *oligobranchiata* [2], and well developed among those of *L*. *paivai* **sp. nov.**

Prebranchial chaetigers of specimens of *Levinsenia paivai* **sp. nov.** were observed in two different states; (1) when dorsal side of prebranchial chaetigers is not inflated, a longitudinal mid-dorsal groove is formed (Fig 2A); (2) when dorsal side of prebranchial chaetigers is inflated, a dorsal crest is formed (Fig 2C). We believe this difference is due to the moment of fixation of the specimen. Probably the movements of an inflated anterior region would help the worm burrow through the sediment.

*Etymology*. This species is named after Dr. Paulo Cesar de Paiva, a well-known Brazilian polychaetologist, expert on systematics, benthic ecology and marine phylogeography of polychaetes, and also a great professor, who continuously inspires and contributes to the education of biologists, including both first authors of this paper.

**Fig 5:**
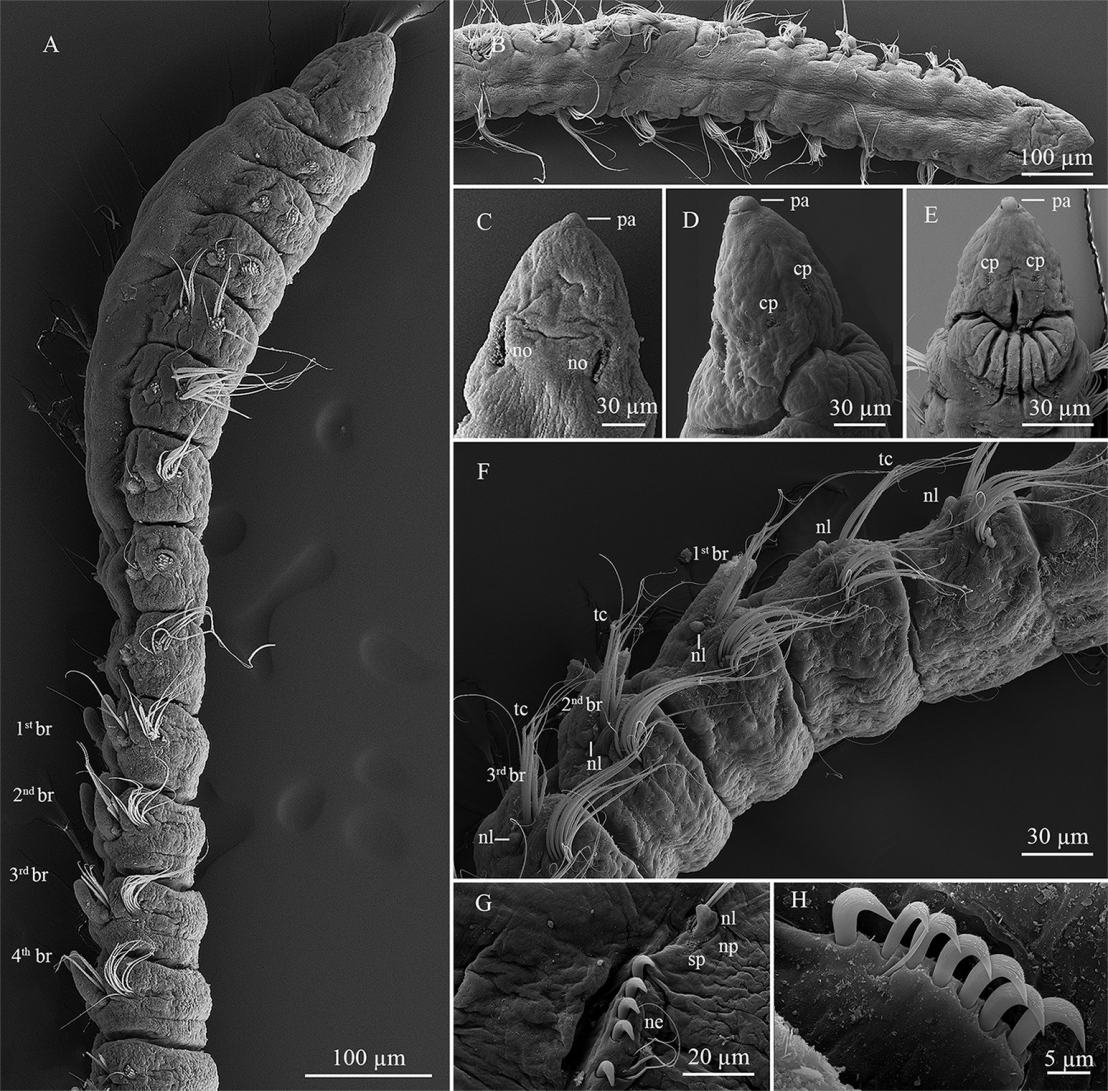
*Levinsenia blakei* sp. nov. (A–B). Anterior end, lateral and dorsal views, respectively; (C–E). Prostomium, dorsal, lateral and ventral views, respectively; (F). Branchial chaetigers, lateral view; (G). Parapodium, chaetiger 36, lateral view; (H). Neuropodium, chaetiger 48. br = branchia; cp = ciliary patches; ne = neuropodium; nl = notopodial postchaetal lobe; no = nuchal organ; np = notopodium; pa = palpode; sp = sensory pores; tc = transitional chaeta.

**Fig 6:**
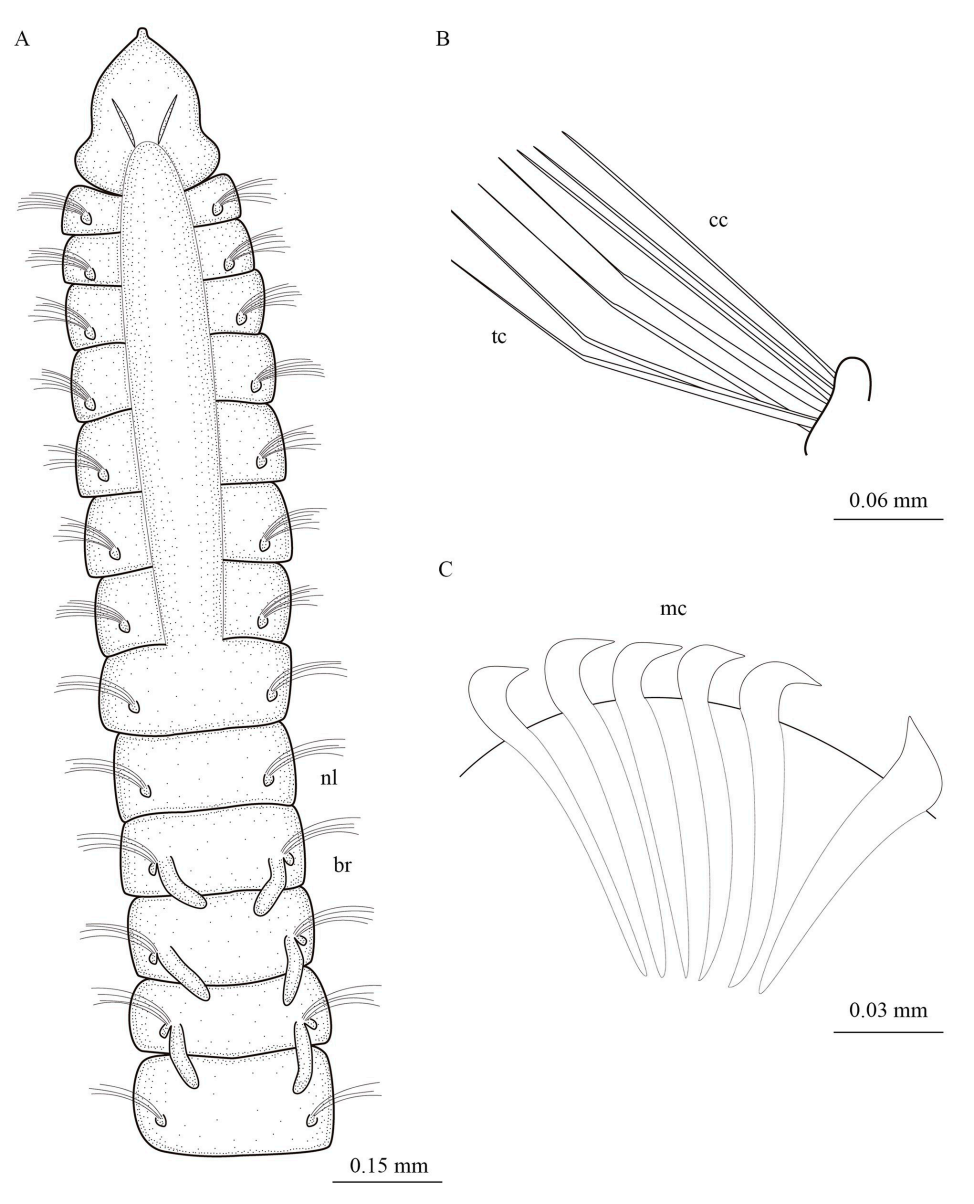
*Levinsenia blakei* sp. nov. (A). Anterior end, dorsal view; (B). Notopodium, chaetiger 8; (C). Neuropodium, chaetiger 38, accompanying capillary chaetae not shown. br = branchia; cc = capillary chaeta; nl = notopodial postchaetal lobe; mc = modified chaeta; tc = transitional chaeta.

*Levinsenia blakei sp*. *nov*. (Figs 4–6)

urn:lsid:zoobank.org:act: 682088DE-C572-44C5-8994-F53F2B654DF1

*Material examined*. *Type series*. Holotype (MZUSP–4152): incomplete, with 47 chaetigers, 9.44 mm long, 0.16 mm wide, coll. 14 Dec 2011, 19°36'26.24"S 39°10'17.35"W, 352 m. Paratypes: 1 spec. (MZUSP–4153), coll. 19 Jun 2013, 20°17'37.38"S 39°42'36.72"W, 1,347 m; 1 spec. (MZUSP–4154), coll. 25 Jun 2013, 19°33'22.17"S 39°02'36.03"W, 446 m; 1 spec. (MZUSP–4155), coll. 28 Jun 2013, 19°49'36.9"S 39°35'42.69"W, 363 m; 1 spec. (ZUEC–21461), coll. 19 Jun 2013, 20°15'32.18"S 39°46'12.38"W, 1,029 m. Specimens mounted on SEM stubs: 1 spec. (MZUSP–4282), coll. 9 Jan 2012, 20°14'19.45"S 39°48'36.67"W, 416 m; 3 specs. (MZUSP–4283), coll. 11 Dec 2011, 19°33'20.99"S 39°02'36.2"W, 374 m.

*Additional material examined*. 1 spec. (MZUSP–4156), coll. 27 Jun 2013, 19°50'06.01"S 39°26'34.62"W, 1,048 m; 1 spec. (MZUSP–4157), coll. 19 Jun 2013, 20°15'32.18"S 39°46'12.38"W, 1,029 m.

*Comparative material examined*. *Levinsenia reducta* (Hartman, 1965) [36], Holotype LACM-AHF POLY 653, North Atlantic, Dutch Guiana, northeastern South America, 07°52’N 54°31.5’W to 07°55’N 54°35’W, 520–550 m, dredge, R/V ATLANTIS Sta. Dr 33, coll. Sanders, H., Woods Hole Oceanographic Institution, 25 Apr 1963. Paratype LACM-AHF POLY 654, North Atlantic, Dutch Guiana, northeastern South America, 07°52’N 54°31.5W to 07°55’N 54°35’W, 520–550 m, dredge, R/V ATLANTIS Sta. DR 33, coll. Sanders, H., Woods Hole Oceanographic Institution, 25 Apr 1963. *Levinsenia kirbyorum* Lovell, 2002 [15] Paratype LACM-AHF POLY 2090, Indian Ocean, Thailand, Andaman Sea, Phang Nga Bay, 08°30’N 98°06’E, 41.7 m, muddy sand, Olavsen box corer, R/V Chakratong Tongyai, Sta. E-1/BC, coll. Thai-Danish BIOSHELF Project, Bussarawit, S., Aungtonya, C., 22 Apr 1996

*Description*. All specimens incomplete, holotype with 47 chaetigers (24–50). Body 9.44 mm long (2.45–9.84 mm), 0.16 mm wide (0.16–0.20 mm), fragile, easily broken. Anterior chaetigers dorso-ventrally flattened, short, wider than long until chaetiger 21 (Figs 5A, 5B and 6A); about as long as wide, biannulate and cylindrical from chaetiger 22 until posterior segments. Preserved specimens white–yellowish, without pigmentation patterns.

Prostomium conical, slightly longer [0.18 mm (0.15–0.18 mm)] than wide [0.14 mm (0.12–0.14 mm)]; cylindrical terminal sensory organ (palpode), sometimes everted; cheek organs, eyes and median antenna all absent; patches of cilia present on ventral and lateral sides (Fig 5C–5E); pair of nuchal organs as ciliated longitudinal slits. Anterior buccal lip with 2 longitudinal folds, posterior buccal lip with six longitudinal folds, extending to middle of chaetiger 1 (Fig 5E). Ventral mouth, saclike pharynx everted in some specimens.

First six chaetigers can be dorsally inflated. Prebranchial region with eight (8–9) wider than long chaetigers; notopodial postchaetal lobes short, rounded tubercles. First pair of branchiae starting from chaetiger 9 (9–10); four (3–4) pairs of short [0.11 mm long (0.10–0.12 mm), 0.04 mm wide] cirriform branchiae; cylindrical, blunt tipped, discretely ciliated (Figs 5F and 6A). Notopodial postchaetal lobes short on branchial chaetigers, tubercular (Fig 5F); also tubercular on postbranchial chaetigers. Notopodial sensory pores present throughout (Fig 5G). Neuropodial postchaetal lobes absent.

Parapodia biramous and poorly developed, notochaetae emerging dorso-laterally, neurochaetae originating laterally. Notochaetae of two types: capillary chaetae and transitional notochaetae; on chaetigers 1–7, notopodia with 10 capillary chaetae each, arranged in two rows, from chaetiger 8 until end of branchial segments, notopodia each with three capillary chaetae in anterior row and four transitional chaetae in posterior row (Figs 5F and 6B), postbranchial chaetigers with five capillary chaetae each.

Neurochaetae of prebranchial and branchial chaetigers all capillary. Postbranchial region with two types of neurochaetae: capillary and modified, strongly curved, stout spines, with expanded shaft and fringe on convex side, slightly protruding from neuropodia (Fig 5G). Modified spines first appearing on chaetiger 22. Prebranchial chaetigers with 12 capillary neurochaetae each, arranged in two rows; branchial chaetigers with 16 capillary neurochaetae each, arranged in three rows; postbranchial chaetigers with 4–7 modified spines, thick, strongly curved, with distinct fringe on convex side, arranged in posterior row, and three accompanying capillary chaetae slenderer than capillary chaetae of preceding regions, arranged in anterior row (Figs 5H and 6C). Pygidium unknown.

*Methyl green stain*. Staining pattern conspicuous, as a solid band per segment, on chaetigers 9–21.

*Habitat*. Found in substrates with a high percentage of mud (81%), some sand (18%) and a small percentage of pebbles (1%); water temperature 4–15.7°C; between 363–1,048 m deep.

*Distribution*. Southern Atlantic Ocean: Southeastern Brazil, off Espírito Santo state, 363–1,048 m (Fig 4).

*Remarks*. Members of *L*. *blakei* **sp. nov.** share the presence of more than seven prebranchial segments with the following congeners: *L*. *acutibranchiata* (Strelzov, 1973) [26], *L*. *antarctica* (Strelzov, 1973) [26], *L*. *brevibranchiata* (Strelzov, 1973) [26], *L*. *canariensis* (Brito & Núñez, 2002) [37], *L*. *kirbyorum* Lovell, 2002 [15], *L*. *kosswigi* Çinar, Dagli & Acik, 2011 [16], and *L*. *reducta* (Hartman, 1965) [36].

Out of those, members of all *L*. *acutibranchiata*, *L*. *kirbyorum*, *L*. *kosswigi*, and *L*. *reducta* are readily distinguished from individuals of *L*. *blakei* **sp. nov.** in having more than 10 branchiate chaetigers [2, 15, 16, 36], while those of the new species only have 4 branchiferous segments.

Specimens of *L*. *antarctica* are similar to members of *L*. *blakei* **sp. nov.** in having few branchiferous segments, only three among members of that species, and also in regards to the morphology of branchiae. However, these animals have seven prebranchial segments, while individuals of *L*. *blakei* **sp. nov.** have 8 or 9. In addition, the notopodial postchaetal lobes of the branchiate segments are cirriform among members of *L*. *antarctica* [2], while in specimens of *L*. *blakei* **sp. nov.** those lobes are tubercular.

Members of *L*. *brevibranchiata* differ from members of *L*. *blakei* **sp. nov.** in having six pairs of branchiae and also in the morphology of the modified spines, which are slightly curved, with no expanded shaft in those animals [2], instead of strongly curved, with expanded shafts, as in members of *L*. *blakei* **sp. nov.**

Individuals of *L*. *canariensis*, as said above, have more than seven prebranchial chaetigers. However, those animals have branchiae beginning far more posteriorly, at the posterior part of the body, which is a very unusual branchial distribution for members of this genus [37].

The first six chaetigers of specimens of *Levinsenia blakei* **sp. nov.** were observed in two different states; (1) when dorsal side of first six chaetigers is inflated, a dorsal crest is formed (Figs 5A and 6A); (2) when dorsal side of first six chaetigers is not inflated, a longitudinal mid-dorsal groove is formed (Fig 5B). We believe this difference is due to the moment of fixation of the specimen. As discussed above for *L*. *paivai*, this is probably due to burrowing movements.

*Etymology*. This species is named after Dr. James Blake, a very important researcher on biology and systematics of polychaetes during the last decades, continuously providing valuable contributions to the knowledge of several families of this group, including Paraonidae.

**Fig 7:**
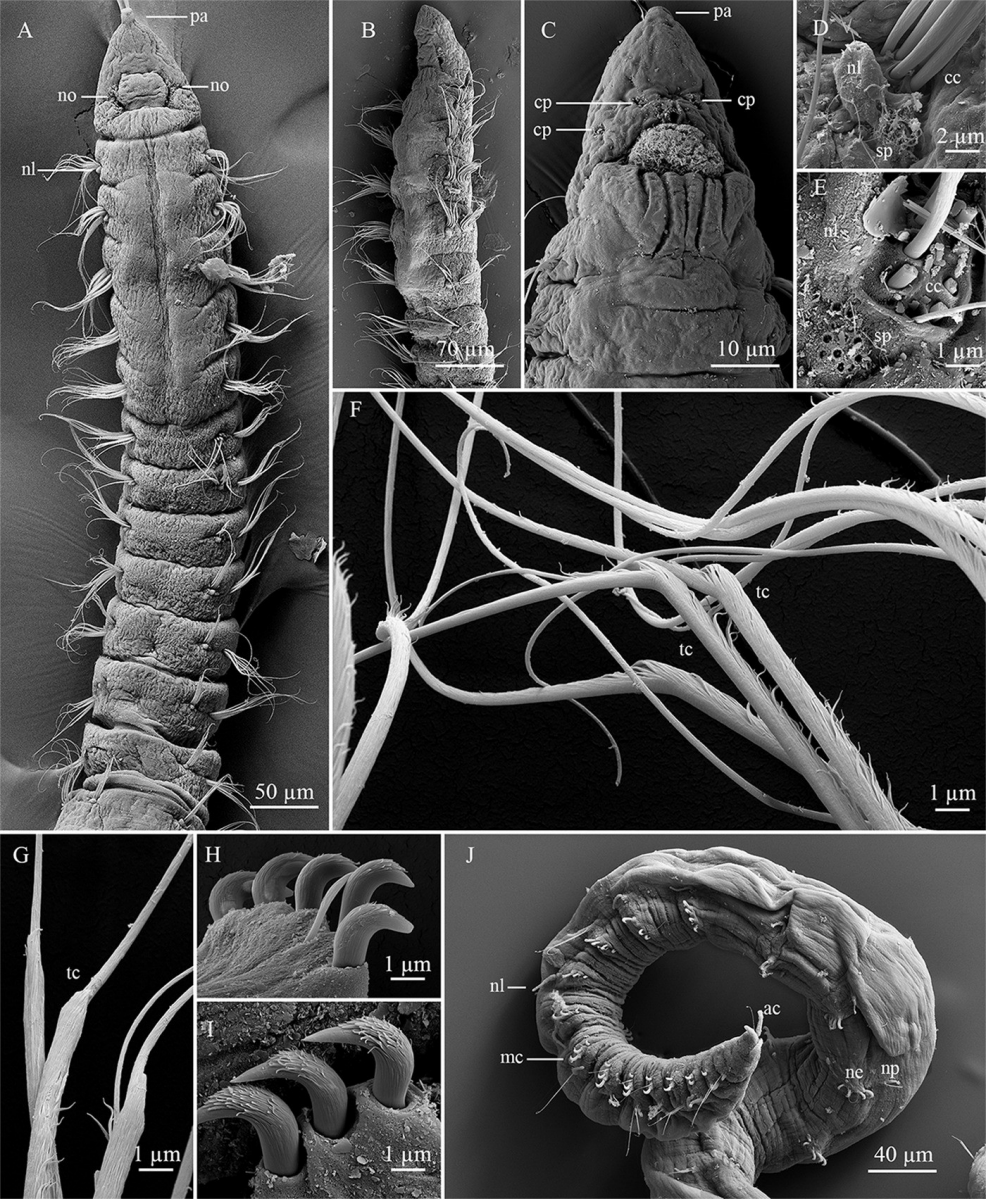
*Levinsenia lesliae* sp. nov. (A–B). Anterior end, dorsal and lateral views, respectively; (C). Prostomium, ventral view; (D). Notopodium, chaetiger 13, lateral view; (E). Notopodium, chaetiger 16, lateral view; (F). Transitional notochaetae, chaetiger 10, lateral view; (G). Detail of transitional chaetae, chaetiger 11, dorsal view (H). Neuropodium, chaetiger 26, lateral view; (I). Neuropodium, chaetiger 42; (J). Posterior end, lateral view. ac = anal cirrus; cc = capilary chaeta; cp = ciliary patches; mc = modified chaeta; ne = neuropodium; nl = notopodial postchaetal lobe; no = nuchal organ; np = notopodium; pa = palpode; sp = sensory pore; tc = transitional chaeta.

**Fig 8:**
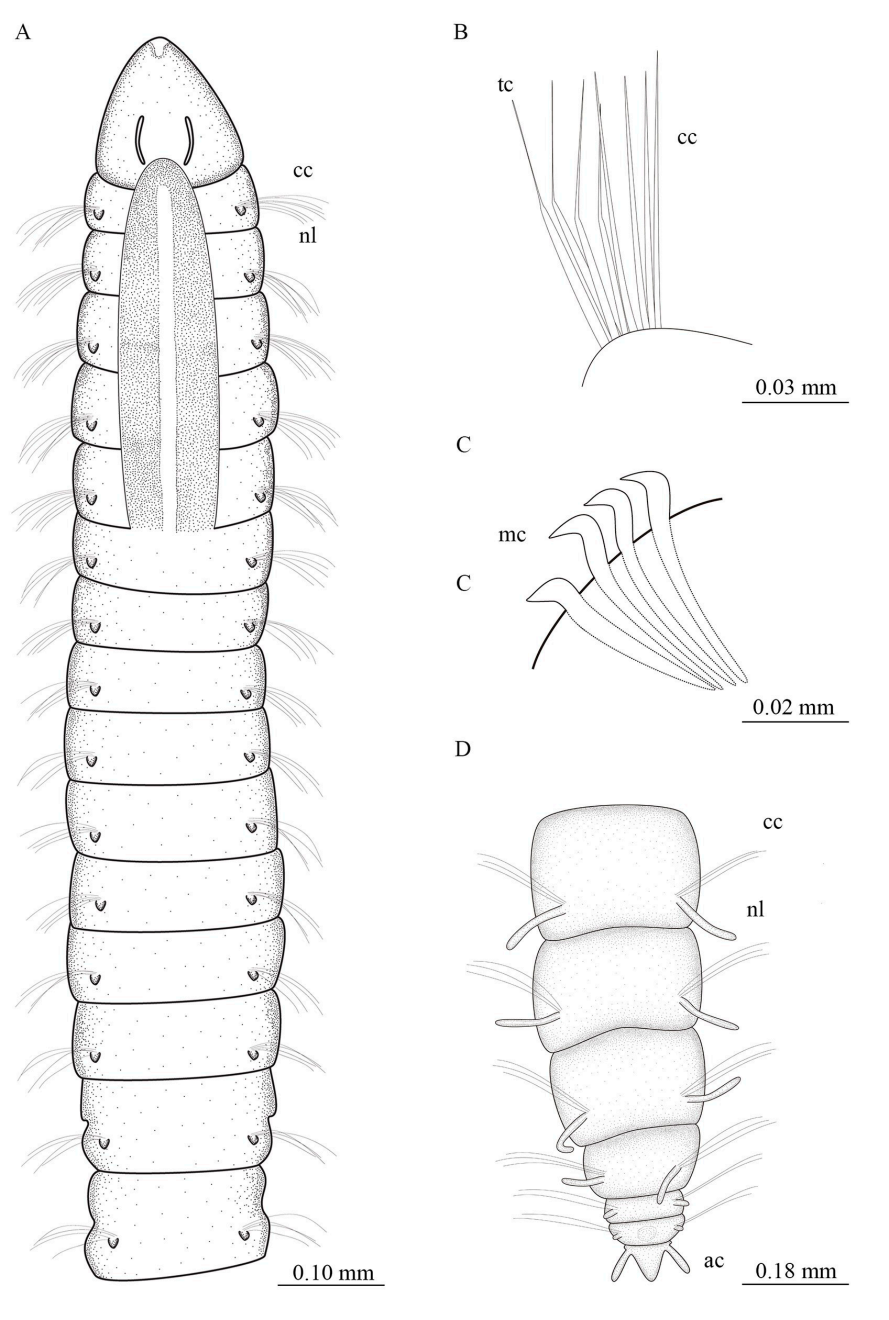
*Levinsenia lesliae* sp. nov. (A). Anterior end, dorsal view; (B). Notopodium, chaetiger 10; (C). Neuropodium, chaetiger 22; (D). Posterior end, dorsal view. ac = anal cirrus; cc = capillary chaeta; mc = modified chaeta; nl = notopodial postchaetal lobe; tc = transitional chaeta.

*Levinsenia lesliae sp*. *nov*. (Figs 4, 7, and 8)

urn:lsid:zoobank.org:act: A3CC98C9-CF5E-41D4-A222-552C5643CA32

*Material examined*. *Type series*. Holotype (MZUSP–4158): 76 chaetigers, complete, 12.62 mm long, 0.16 mm wide, coll. 15 Jan 2012, 19°45'54.56"S 39°30'25.23"W, 121 m; Paratypes: 10 specs. (MZUSP–4159), coll. 23 Jan 2012, 21°04'04.76"S 40°14'14.14"W, 142 m; 10 specs. (MZUSP–4160), coll. 27 Jun 2013, 19°45'53.43"S 39°30'25.97"W, 138 m; 10 specs. (MZUSP–4161), coll. 29 Jun 2013, 19°36'03.57"S 39°10'33.64"W, 142 m; 8 specs. (MZUSP–4162), coll. 9 Dec 2011, 19°31'51.66"S 39°03'04.04"W, 140 m; 10 specs. (MZUSP–4163), coll. 14 Jan 2012, 19°49'7.27"S 39°36'08.52"W, 124 m; 10 specs. (UFBA–1861), coll. 14 Jan 2012, 19°49'07.27"S 39°36'08.52"W, 124 m; 10 specs. (UFBA–1862), coll. 15 Jan 2012, 19°45'55.39"S 39°30'25.74"W, 121 m; 10 specs. (UFBA–1863), coll. 15 Jan 2012, 19°36'04.32"S 39°10'34.07"W, 134 m; 10 specs. (UFBA–1864), coll. 29 Jun 2013, 19°49'06.26"S 39°36'09.34"W, 181 m; 10 specs. (ZUEC–21462), coll. 14 Jan 2012, 19°49'07.27"S 39°36'08.52"W, 124 m; 10 specs. (ZUEC–21463), coll. 15 Jan 2012, 19°45'55.39"S 39°30'25.74"W, 121 m; 10 specs. (ZUEC–21464), coll. 15 Jan 2012, 19°36'04.32"S 39°10'34.07"W, 134 m; 10 specs. (ZUEC–21465), coll. 29 Jun 2013, 19°49'06.26"S 39°36'09.34"W, 181 m; 1 spec. (ZUEC–21466), coll. 30 Jun 2013, 19°03'39.78"S 37°47'39.35"W, 1,874 m. Specimens mounted on SEM stubs: 2 specs. (ZUEC–21467), coll. 14 Jan 2012, 19°49'07.27"S 39°36'08.52"W, 124 m; 2 specs. (ZUEC–21468), coll. 21 Jan 2012, 20°34'32.47"S 40°20'52.37"W, 20 m.

*Additional material*. 38 specs. (MZUSP–4164), coll. 23 Jan 2012, 21°04'04.76"S 40°14'14.14"W, 142 m; 1 spec. (MZUSP–4165), coll. 30 Dec 2011, 21°04'09.61"S 40°13'07.38"W, 396 m; 19 specs. (MZUSP–4166), coll. 21 Jan 2012, 20°34'32.47"S 40°20'52.37"W, 20 m; 44 specs. (MZUSP–4167), coll. 8 Jan 2012, 20°35'16.23"S 39°53'47.1"W, 382 m; 30 specs. (MZUSP–4168), coll. 20 Jan 2012, 20°11'25.35"S 40°02'16.02"W, 35 m; 57 specs. (MZUSP–4169), coll. 9 Jan 2012, 20°14'19.45"S 39°48'36.67"W, 416 m; 3 specs. (MZUSP–4170), coll. 9 Dec 2011, 19°31'51.66"S 39°03'04.04"W, 140 m; 7 specs. (MZUSP–4171), coll. 11 Dec 2011, 19°33'20.99"S 39°02'36.2"W, 384 m; 2,719 specs. (MZUSP–4172), coll. 14 Jan 2012, 19°49'07.27"S 39°36'08.52"W, 124 m; 2 specs. (MZUSP–4173), coll. 14 Jan 2012, 19°49'37.21"S 39°35'41.25"W, 352 m; 1 spec. (MZUSP–4174), coll. 13 Jan 2012, 19°53'31.53"S 39°32'56.35"W, 955 m; 110 specs. (MZUSP–4175), coll. 15 Jan 2012, 19°45'55.39"S 39°30'25.74"W, 121 m; 28 specs. (MZUSP–4176), coll. 14 Jan 2012, 19°46'34.99"S 39°30'04.65"W, 402 m; 8 specs. (MZUSP–4177), coll. 11 Jan 2012, 19°50'01.87"S 39°26'30.04"W, 1,053 m; 1 spec. (MZUSP–4178), coll. 19 Jan 2012, 19°26'05"S 39°17'38.92"W, 46 m; 35 specs. (MZUSP–4179), coll. 15 Jan 2012, 19°36'04.32"S 39°10'34.07"W, 134 m; 35 specs. (MZUSP–4180), coll. 14 Dec 2011, 19°36'26.24"S 39°10'17.35"W, 352 m; 1 spec. (MZUSP–4181), coll. 13 Dec 2011, 19°40'08.03"S 39°7'22.1"W, 1,010 m; 7 specs. (MZUSP–4182), coll. 18 Jan 2012, 18°53'29.72"S 39°06'23.3"W, 43 m; 3 specs. (MZUSP–4183), coll. 12 Dec 2010, 19°41'33.92"S 39°31'17.74"W, 48 m; 3 specs. (MZUSP–4184), coll. 17 Jan 2012, 18°40'55.3"S 38°55'41.48"W, 44 m; 18 specs. (MZUSP–4185), coll. 11 Jul 2013, 21°04'04.56"S 40°14'14.08"W, 147 m; 2 specs. (MZUSP–4186), coll. 9 Jun 2013, 21°04'43.08"S 40°04'12.96"W, 1,331 m; 38 specs. (MZUSP–4187), coll. 12 Jul 2013, 20°34'34.37"S 40°20'50.77"W, 21 m; 5 specs. (MZUSP–4188), coll. 13 Jul 2013, 20°35'23.09"S 39°55'01.18"W, 156 m; 1 spec. (MZUSP–4189), coll. 13 Jul 2013, 20°11'25.75"S 40°02'15.87"W, 33 m; 1 spec. (MZUSP–4190), coll. 13 Jul 2013, 20°12'21.46"S 39°58'0.3"W, 45 m; 23 specs. (MZUSP–4191), coll. 19 Jun 2013, 20°14'17.95"S 39°48'34.35"W, 418 m; 47 specs. (MZUSP–4192), coll. 29 Jun 2013, 19°31'51.68"S 39°03'04.79"W, 163 m; 4 specs. (MZUSP–4193), coll. 25 Jun 2013, 19°33'22.17"S 39°02'36.03"W, 446 m; 5 specs. (MZUSP–4194), coll. 25 Jun 2013, 19°37'45.14"S 39°03'58.75"W, 1,036 m; 1,429 specs. (MZUSP–4195), coll. 29 Jun 2013, 19°49'06.26"S 39°36'09.34"W, 181 m; 11 specs. (MZUSP–4196), coll. 28 Jun 2013, 19°49'36.9"S 39°35'42.69"W, 363 m; 43 specs. (MZUSP–4197), coll. 27 Jun 2013, 19°45'53.43"S 39°30'25.97"W, 138 m; 49 specs. (MZUSP–4198), coll. 27 Jun 2013, 19°46'32.84"S 39°30'03.65"W, 431 m; 1 spec. (MZUSP–4199), coll. 14 Jul 2013, 19°26'04.81"S 39°17'38.64"W, 43 m; 30 specs. (MZUSP–4200), coll. 29 Jun 2013, 19°36'03.57"S 39°10'33.64"W, 142 m; 13 specs. (MZUSP–4201), coll. 26 Jun 2013, 19°36'30.6"S 39°10'19.39"W, 349 m; 2 specs. (MZUSP–4202), coll. 13 Jun 2013, 20°46'17.79"S 38°17'16.01"W, 3,020 m; 18 specs. (MZUSP–4203), coll. 16 Jul 2013, 18°53'31.97"S 39°06'21.78"W, 43 m; 1 spec. (MZUSP–4204), coll. 16 Jul 2013, 19°33'02.92"S 38°42'52.26"W, 138 m; 12 specs. (MZUSP–4205), coll. 14 Jun 2013, 19°52'52.59"S 38°35'10.48"W, 1,010 m; 2 specs. (MZUSP–4206), coll. 14 Jun 2013, 20°04'09.68"S 38°31'29.01"W, 1,287 m; 2 specs. (MZUSP–4207), coll. 14 Jun 2013, 20°16'38.17"S 38°27'26.52"W, 1,897 m; 4 specs. (MZUSP–4208), coll. 13 Jun 2013, 20°29'03.85"S 38°23'18.56"W, 2,500 m; 1 spec. (MZUSP–4209), coll. 17 Jul 2011, 20°01'02.6"S 39°50'18.72"W, 49 m; 1 spec. (MZUSP–4210), coll. 13 Jul 2011, 19°41'24.99"S 39°31'20.42"W, 44 m; 1 spec. (MZUSP–4211), coll. 23 Jun 2013, 19°03'10.22"S 37°45'28.45"W, 2,823 m; 7 specs. (MZUSP–4212), coll. 2 Jul 2013, 18°40'57.41"S 38°55'39.92"W, 44 m; 3 specs. (MZUSP–4213), coll. 1 Jul 2013, 19°03'30.62"S 37°48'46.66"W, 1,361 m; 2 specs. (MZUSP–4214), coll. 30 Jun 2013, 19°03'39.78"S 37°47'39.35"W, 1,874 m; 3 specs. (MZUSP–4215), coll. 23 Jun 2013, 19°03'10.76"S 37°45'34.37"W, 2,664 m.

*Comparative material examined*. *Levinsenia uncinata* (Hartman, 1965) [36] Holotype LACM-AHF POLY 657 North Atlantic, Bermuda, Bermuda slope, 32°14’18”N 64°42’W, 1,500 m, R/V ATLANTIS Sta. Bermuda 6, coll. Sanders, H., Woods Hole Oceanographic Institution, 1 Sep 1961. Paratype LACM-AHF POLY 658, North Atlantic, Bermuda, Bermuda slope, 32°14’18”N 64°42’W, 1,500 m, R/V ATLANTIS Sta. Bermuda 6, coll. Sanders, H., Woods Hole Oceanographic Institution, 1 Sep 1961.

*Description*. Holotype complete with 76 chaetigers (44–82). Body 12.62 mm long (4.00–12.88 mm), 0.16 mm wide (0.15–0.21 mm), fragile, easily broken. Anterior chaetigers dorso-ventrally flattened, much wider than long (Figs 7A and 8A); from chaetiger 14 to posterior body, segments about as long as wide, biannulate and cylindrical; last 12 chaetigers wider than long. Preserved specimens white–yellowish, without pigmentation patterns.

Prostomium conical, slightly longer [0.16 mm (0.09–0.19 mm)] than wide [0.10 mm (0.08–0.14 mm)]; cylindrical terminal sensory organ (palpode), sometimes everted (Fig 7A and 7B); cheek organs, eyes and median antenna all absent; patches of ciliation on ventral and lateral sides (Fig 7C); pair of nuchal organs as ciliated longitudinal slits. Anterior buccal lip with two longitudinal folds, posterior buccal lip with four longitudinal folds, extending to middle of chaetiger 1 (Fig 7C). Ventral mouth with saclike ciliated pharynx, everted in some specimens.

First five chaetigers sometimes dorsally inflated (Fig 7B). Notopodial postchaetal lobes tubercular on all anterior segments (Figs 7D, 7E and 8A), filiform in last 20 chaetigers (Figs 7J and 8D), 0.04 mm long. Notopodial sensory pores present along entire body (Fig 7E), with projecting filament (Fig 7D). Neuropodial postchaetal lobes absent throughout. Branchiae absent (Figs 7A and 8A). Parapodia biramous and poorly developed, notochaetae emerging dorso-laterally, neurochaetae originating laterally. Notopodia with two types of chaetae: capillary and transitional chaetae (Figs 7F, 7G and 8B); notopodia of first five chaetigers with 12 capillary chaetae each, arranged in two rows, on chaetigers 6–8, notopodia each with 8 capillary chaetae, arranged in two rows, on chaetigers 9–12, 4 transitional chaetae per notopodium and 4 capillary chaetae, arranged in two rows, from chaetiger 13 to end of body, notopodia with 5 capillary chaetae each, arranged in two rows.

Neuropodia with two types of chaetae, capillary chaetae and modified neuropodial spines (Figs 7H, 7I and 8C), starting on chaetiger 14 (12–15). Neuropodia of first five chaetigers with 12 capillary chaetae each, arranged in two rows; on chaetigers 6–12, neuropodia with 8 capillary chaetae each, arranged in two rows; from segment 14 to end of body, neuropodia each with 3–6 modified stout spines, strongly curved, with expanded shaft and distinct fringe on convex side, arranged in a single row (Fig 7H–7J), and 3 accompanying capillary chaetae slenderer than capillary chaetae of preceding regions. Pygidium conical, slightly longer than wide, with one pair of anal cirri 0.05 mm (0.04–0.06) mm long (Figs 7J and 8D).

*Methyl green stain*. First five chaetigers weakly staining, only on postchaetal areas; on chaetigers 6–12, strong staining pattern, as a solid band on each segment.

*Habitat*. Summer: Found in substrates with high percentage of mud (52%) and sand (46%), and some pebbles (2%), water temperature 2.6–26°C; between 20–3,020 m deep.

*Distribution*. Southern Atlantic Ocean: Southeastern Brazil, off Espírito Santo state, 20–3,020 m (Fig 4).

*Remarks*. The absence of branchiae is a rare character among species of *Levinsenia*, being known only from members of *L*. *hawaiiensis* Giere, Ebbe & Erséus, 2007 [38] and *L*. *uncinata* (Hartman, 1965) [36], besides *L*. *lesliae* **sp. nov.**

Members of *L*. *hawaiiensis* are easily distinguished from specimens of *L*. *lesliae* **sp. nov.**, as they have bifid modified neuropodial spines and three pygidial cirri, instead of a pair. Both these characters are unusual for members of *Levinsenia* and it is debatable whether the species belongs in this genus [10].

Members of *L*. *uncinata*, in addition to the absence of branchiae, closely resemble those of *L*. *lesliae* **sp. nov.** in having tubercular notopodial postchaetal lobes on anterior chaetigers, and on the beginning of the modified spines, chaetiger 16 among members of *L*. *uncinata*, chaetiger 14 in specimens of *L*. *lesliae* **sp. nov.** Members of these species differ, however, because individuals of *L*. *uncinata* lack notopodial transitional chaetae and their neuropodial modified spines strongly protrude from neuropodia and are slightly curved [2, 36], while among members of *L*. *lesliae* **sp. nov.** those spines barely protrude from parapodia and are strongly curved, with expanded shafts.

The first five chaetigers of specimens of *Levinsenia lesliae* **sp. nov.** were observed in two different states; (1) when dorsal side of first five chaetigers is not inflated a longitudinal mid-dorsal groove is formed (Figs 7A and 8A); (2) when dorsal side of first five chaetigers is inflated, a dorsal crest is formed (Fig 7B). We believe this difference is due to the moment of fixation of the specimen and reflects the burrowing movements of the worm, as discussed for both the species described above.

Although *Levinsenia lesliae* **sp. nov.** was found in a wide bathymetric range there is a considerable difference in their distribution between summer and winter samples. In summer samples, specimens were found between 20–1,053 m deep, even though samples were taken until 3,000 m deep. During the winter, specimens have a wider bathymetric range (20–3,020 m deep), being noticeable that only in two, out of the eight slope transects, individuals of *L*. *lesliae* **sp. nov.** were found below 1,054 m. Furthermore, few specimens were found in those deeper stations, totaling only 21 individuals collected below 1,054 m (Table 2). A careful morphological examination was performed among individuals from shallower (until ~1,000 m deep) and deeper (below ~1,000 m deep) stations, but no significant difference was observed.

**Table 2:** Abundance of *Levinsenia lesliae* sp. nov. at different bathymetric ranges.

	20–49 m	50–181 m	182–1053 m	1054–3020 m
**Summer**	60	3006	184	0
**Winter**	68	1613	117	21

*Etymology*. This species is named after Leslie Harris, for all her contributions to the knowledge of polychaetes. An extraordinarily kind person, who made possible for the first author to visit the Polychaete Collection of the Natural History Museum of Los Angeles County.

## Discussion

The three new species of *Levinsenia* described in this paper showed different patterns of bathymetric distribution. Individuals of *L*. *paivai* **sp. nov.** have a shallow and restricted bathymetric range, not exceeding 150 m deep. A wider bathymetric range was observed for members of *L*. *blakei* **sp. nov.**, which were found between 300–1,400 m, and even greater among members of *L*. *lesliae* **sp. nov.**, which were collected between 20–3,020 m. Members of this latter species also showed the highest abundance, with 3,250 specimens collected during summer and 1,819 during winter; these animals were found in all the four sampling areas, with a remarkably high abundance at a particular spot in canyon Watu Norte, from which 2,749 specimens were obtained in summer and 1,449 in winter.

Recently, due to the modern techniques available, especially SEM, new morphological characters have been added to the genus *Levinsenia*, such as the patches of ciliation on the prostomium; notopodial sensory pores; bands of cilia across the dorsum, connecting branchiae within pairs; and details of pubescence on modified neurochaetae [18]. In addition to these characters, methyl green staining patterns seem to be a consistent species specific character [15, 18], showing consistent differences between members of the three species on the distribution of chaetigers stained with a solid band.

Methyl green staining has become an useful tool for the systematics at species level in several groups of polychaetes, such as Spionidae, Capitellidae, Cirratulidae, Sabellidae and Terebelliformia [39–43]. We suggest a similar search for new morphological characters and new techniques, such as the use of methyl green, should be carried out for the other genera of Paraonidae.

This study is also important due to the area sampled, and when it was made. The region was strongly impacted by the collapse of a mining damn inland in Brazil, which released over 50 million cubic meters of mining tailings into Rio Doce, by the end of 2015 [44]. Those mining tailings ended up in the Atlantic through the estuary of Rio Doce, spreading all over the bottom of Espírito Santo basin [44], precisely the area investigated for this study. As the collections for this study were made before that event, it is a possible that the species described herein have become locally extinct, making it fundamental at this point to have a better knowledge of the fauna previously occurring in the area, to properly evaluate the impacts of that disaster. Decreases in diversity and species eveness of macrofaunal assemblages have already been observed following the accident [45].
